# Requirement of LIM domains for the transient accumulation of paxillin at damaged stress fibres

**DOI:** 10.1242/bio.20134531

**Published:** 2013-05-23

**Authors:** Takahiro Watanabe-Nakayama, Masakazu Saito, Shin'ichi Machida, Kikuo Kishimoto, Rehana Afrin, Atsushi Ikai

**Affiliations:** 1 Innovation Laboratory, Tokyo Institute of Technology, S2-8, 4259 Nagatsuta-cho, Midori-ku, Yokohama 226-8503, Japan; 2 Graduate School of Engineering, Tokyo Institute of Technology, I6-1, 2-12-1 Ookayama, Meguro-ku, Tokyo 152-8550, Japan; * Present address: Imaging Research Division, Bio-AFM Frontier Research Center, College of Science and Engineering, Kanazawa University, Kakuma-machi, Kanazawa 920-1192, Japan

**Keywords:** Repair of stress fibres, Mechanosensors, LIM domains

## Abstract

Cells recognize and respond to changes in intra- and extracellular mechanical conditions to maintain their mechanical homeostasis. Linear contractile bundles of actin filaments and myosin II known as stress fibres (SFs) mediate mechanical signals. Mechanical cues such as excessive stress driven by myosin II and/or external force may damage SFs and induce the local transient accumulation of SF-repair complexes (zyxin and VASP) at the damaged sites. Using an atomic force microscope mounted on a fluorescence microscope, we applied mechanical damage to cells expressing fluorescently tagged cytoskeletal proteins and recorded the subsequent mobilization of SF-repair complexes. We found that a LIM protein, paxillin, transiently accumulated at the damaged sites earlier than zyxin, while paxillin knockdown did not affect the kinetics of zyxin translocation. The C-terminal half of paxillin, comprising four-tandem LIM domains, can still translocate to damaged sites on SFs, suggesting that the LIM domain is essential for the mechanosensory function of paxillin. Our findings demonstrate a crucial role of the LIM domain in mechanosensing LIM proteins.

## Introduction

The ability to sense and respond to intra- and extracellular mechanical load is vital for many types of living cells to maintain their normal physiology. Cells can regulate and change their mechanical properties (stiffness, shape and viscoelasticity) and gene expression via mechanosensory and mechanochemical transduction systems in response to mechanical stimuli from their surroundings (Wang et al., 2009). Recent clinical studies have also disclosed new mechanical response systems in many types of cells associated with diseases such as aneurysm, cardiac hypertrophy, and tumor progression (Hahn and Schwartz, 2009; Jaalouk and Lammerding, 2009). Basic knowledge of the mechanisms underlying mechanosensory and mechanochemical transduction systems in living cells is emerging, and is key to understanding how living systems maintain their homeostasis and modulate their mechanical and molecular properties.

The actin cytoskeleton is the main determinant of the mechanical properties of cells, and it plays a role in transmitting mechanical signals throughout the cell. Actin filaments in adherent cells form linear contractile bundles called stress fibres (SFs). SFs have a highly ordered side-by-side alignment of actin filaments non-covalently cross-linked by α-actinin and the motor protein, myosin II. SFs can be induced through the Rho signaling pathway and are often linked to the extracellular matrix (ECM) via focal adhesions (FAs), which include the integrin family of transmembrane ECM receptors (Tojkander et al., 2012). This transmembrane network comprising SFs, FAs and ECM enables bidirectional communication across the cell membrane. First, the inside-out signaling of changes in the actomyosin-dependent contractile force in SFs is transmitted to the ECM and plays critical roles in cell motility, ECM remodeling and tissue morphogenesis (Harris et al., 1980; Lauffenburger and Horwitz, 1996; Wang et al., 1993). Second, reduction in tensile force on SFs produced by inhibitors of myosin II or the Rho signaling pathway causes SF thinning and dissociation of FA proteins (Hotulainen and Lappalainen, 2006). In contrast, outside-in signaling, in which information on ECM rigidity is transmitted to SFs, plays a leading role in tissue remodeling and differentiation. It is also known that the application of external strains and stresses produces FA reinforcement (Riveline et al., 2001), SF thickening and SF reorientation (Yoshigi et al., 2005). The formation and dissociation of SFs and FAs are thus drastically changed depending on intra- and extracellular mechanical conditions. As such, the SF-FA system itself is a bidirectional mechanochemical transduction network.

The results of recent studies suggest several candidates for mechanosensing proteins in FAs and propose a mechanism by which these proteins convert mechanical signals into chemical ones. For example, the expression levels of the three proteins, paxillin, focal adhesion kinase (FAK) and p130Cas, in FAs increase in response to the application of stretching force (Sawada and Sheetz, 2002). Mechanical extension of some FA molecules such as p130Cas or talin modulates their affinity to their partner proteins (Sawada et al., 2006; del Rio et al., 2009). The LIM protein, zyxin, is a focal adhesion protein that exhibits mechanosensory properties.

The intracellular localization of zyxin changes in response to mechanical cues, i.e., zyxin localization at FAs depends on the myosin-mediated tensile force on SFs (Hirata et al., 2008; Lele et al., 2006). Zyxin is also associated with the load-dependent dynamics of SFs, such as their reorientation in response to cyclic stretching (Yoshigi et al., 2005) and actin flux in FAs on rigid substrates (Guo and Wang, 2007). Zyxin translocates to severed or damaged sites on SFs (Colombelli et al., 2009) and recruits the phosphoprotein VASP (Smith et al., 2010), a focal adhesion protein which promotes actin polymerization (Hansen and Mullins, 2010). Finally, α-actinin is recruited to the repair site (Smith et al., 2010). In this way, the zyxin-VASP system repairs the damaged SFs. However, the mechanism underlying the assembly and disassembly of the proteins involved in this process remains to be characterized.

Here, we explored the roles of α_V_-integrin, talin and paxillin as candidate proteins involved in the repair of SFs. These proteins are implicated in the process of FA maturation upstream of zyxin (Zaidel-Bar et al., 2003). We applied an external load to fluorescently labeled SFs in rat fibroblasts using an atomic force microscope (AFM) mounted on a fluorescence microscope. We found that as well as zyxin, paxillin, but not α_V_-integrin or talin, accumulates at damaged sites on SFs to mediate their repair. Kinetic analysis revealed that the accumulation and dissociation of paxillin occurred slightly earlier than zyxin. In contrast, the accumulation and dissociation rates of zyxin at the damaged sites were not affected by the accumulation of paxillin, suggesting that transient accumulation of zyxin was independent of paxillin. Furthermore, we found that a recombinant C-terminal fragment of paxillin comprising only the LIM domains exhibited a similar transient accumulation and dissociation pattern to that of paxillin. These results suggest that the LIM domains are essential for the mechanochemical coupling mechanism of mechanosensory LIM proteins.

## Results and Discussion

### Stress fibre repair following AFM manipulation

**Fig. 1.**
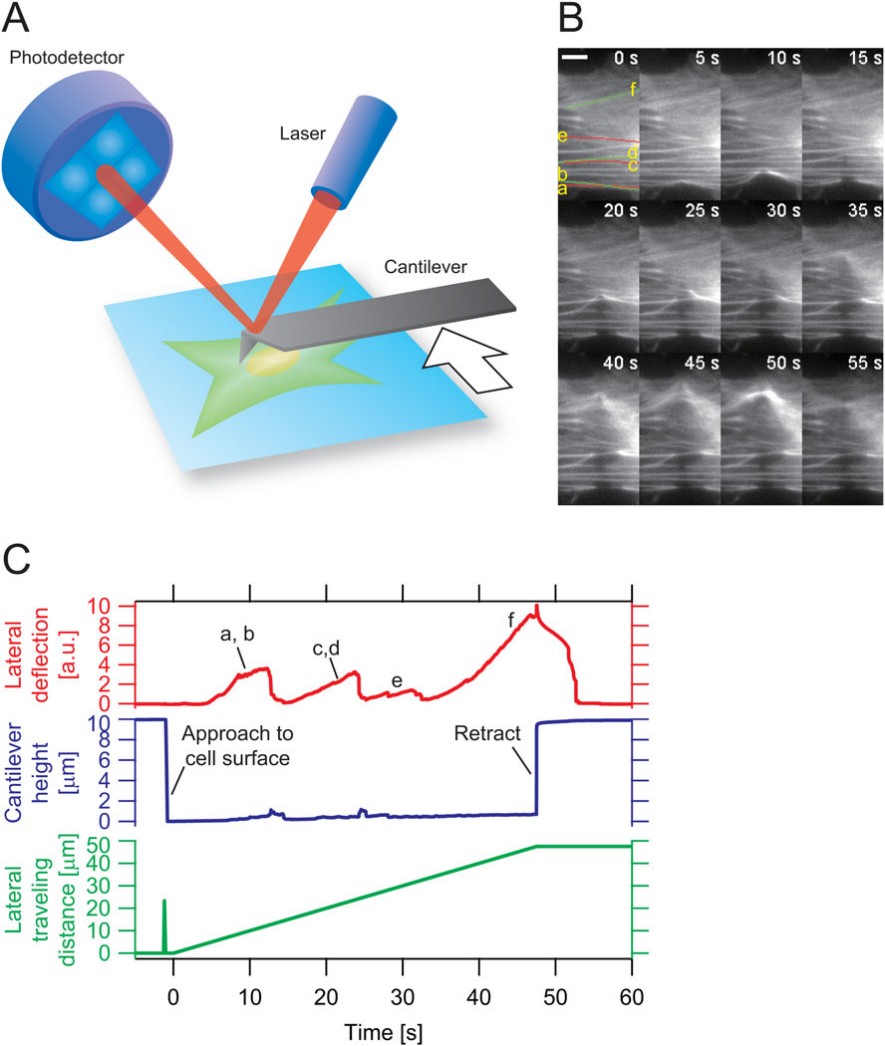
Creation of localized mechanical damage on SFs by AFM manipulation. (**A**) Experimental design for the application of lateral force to SFs. The rat fibroblast cell line, VNOf, transiently coexpressing AcGFP1-actin and TagRFP-focal adhesion protein, was cultured on fibronectin-coated glass slips. The probe tip of the AFM cantilever pushed the cell in close proximity to one of the fluorescently visualized SFs. The cantilever was then moved sideways, pushing and straining the SFs in the lateral direction. (**B**) Time-lapse micrographs of SFs before (0 s) and during (5–55 s) lateral force application. The major SFs (a–f) were strained by lateral travel of the probe tip. The entire process was also recorded as lateral deflection of the cantilever (**C**). The strained SFs underwent thinning from the damaged sites. Scale bar: 10 µm.

**Fig. 2.**
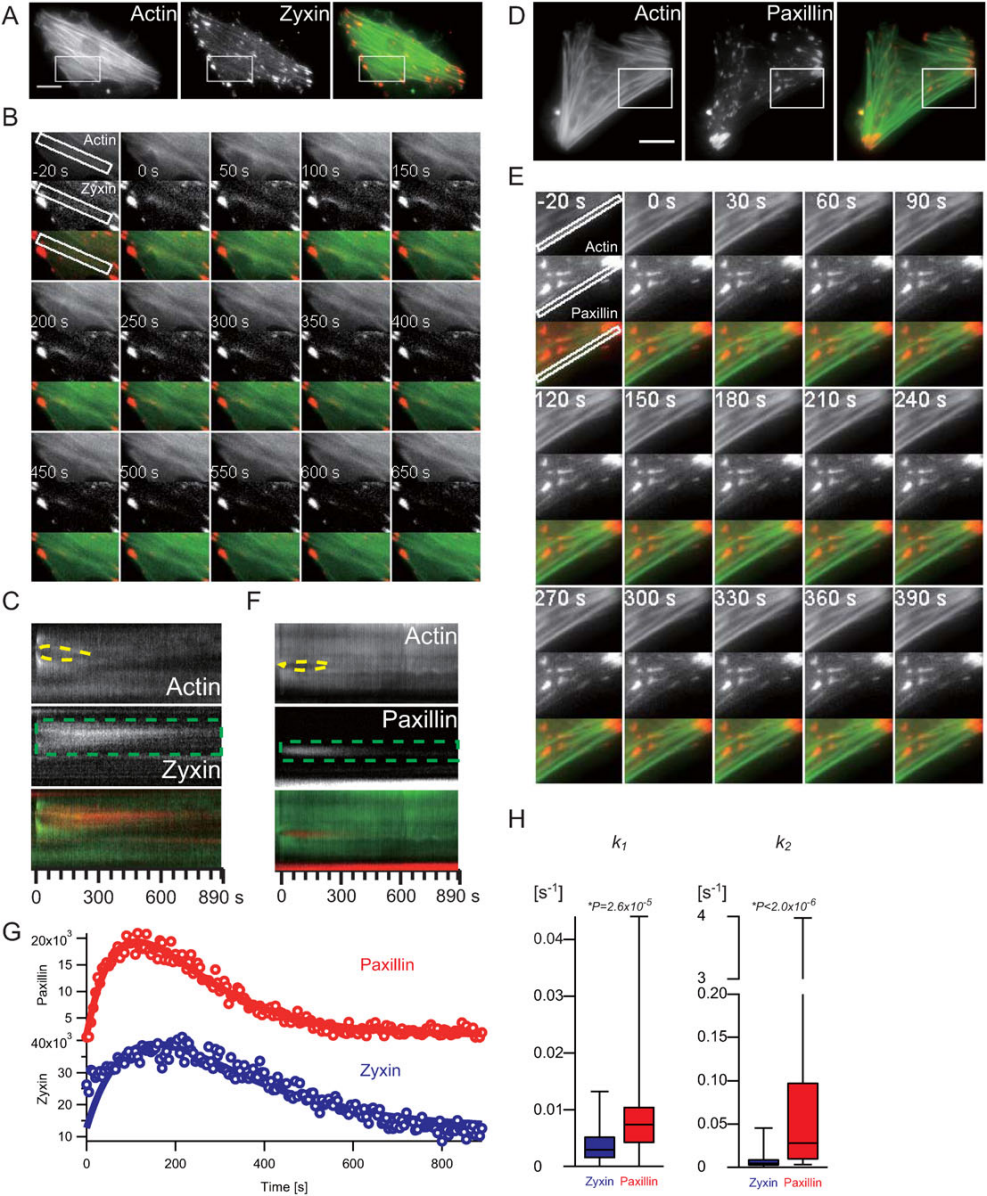
Repair of damaged SFs. The micrographs, the montage images and the kymographs are of AcGFP1-actin and TagRFP-adhesion proteins (**A–C**: zyxin, **D–F**: paxillin) in rat fibroblasts. The montage images (B,E) were taken from the white boxes in the micrograghs (A,D). The kymographs (C,F) from the white boxes in the montage images clearly show paxillin and zyxin accumulation at the region of SF strain (dashed green boxes). The dashed yellow lines in the kymographs show the strained SFs undergoing thinning, elongation, then repair. Scale bars: 20 µm. (**G**) Changes in the fluorescence intensity (open circles) of zyxin-TagRFP (blue) and TagRFP-paxillin (red) after the application of mechanical damage to an SF as estimated from the kymographs (C,F, respectively) and fitted with a simple successive reaction model (solid lines) as described in Materials and Methods. (**H**) Distributions of kinetic parameters for paxillin (red) and zyxin (blue) accumulation and dissociation during repair of damaged stress fibres as box plots of the median values of each parameter. *P*-value represents the probability that the two distributions are indistinguishable according to the Mann–Whitney test. All parameters were estimated from the kymographs (zyxin: 21 cells, 52 positions of 50 SFs; paxillin: 16 cells, 33 SFs).

To understand how SFs in living cells maintain their nearly constant size and shape (homeostasis) under stressful *in vivo* conditions, we recorded the recovery of a single SF labeled with AcGFP1 in a rat fibroblast after applying mechanical damage to the fibre by AFM manipulation. First, the tip end of an AFM cantilever was placed at the side of an SF fluorescently labeled with AcGFP1. The cantilever was then moved laterally while keeping a finite angle to the fibre using the slow feedback control of the z-piezo module (Fig. 1A). During the lateral movement of the cantilever, the AFM tip came into contact with SFs one after another (Fig. 1B). Lateral deflection of the cantilever increased as it pushed an SF, which became deformed and damaged (Fig. 1C). Occasionally, the AFM tip completely cut an SF and then released it with a coincident decrease in the lateral deflection of the cantilever. This process was repeated for every encounter of the tip with SFs. The fluorescence intensity from AcGFP1-actin at a partially cut SF site displayed a sequential response composed of two phases: rapid thinning and elongation of the fibre at the damaged locale followed by gradual thickening of the SF leading to apparent recovery (Fig. 2B,C,E,F) from the damage. We thus demonstrated that cells can repair damaged SFs using their autonomous SF-repair system and maintain their internal mechanical homeostasis. This observation is consistent with the events that follow the spontaneous breakage of SFs driven by myosin II (Smith et al., 2010). Although there have been reports, for example, which demonstrate a drastically altered SF kinetics in EGFP-actin cells (Deibler et al., 2011), the restoration of actin at SF damaged sites was observed using either fluorescence protein-tagged actin or rhodamine-actin (Smith et al., 2010).

### Transient zyxin accumulation at damaged sites on SFs

Smith et al. reported that the focal adhesion LIM protein, zyxin, transiently accumulated at damaged sites on SFs and recruited the actin-polymerizing factor, VASP (Smith et al., 2010). We also confirmed the transient accumulation of zyxin at damaged sites on SFs as described above.

We then followed the accumulation of zyxin at damaged sites on SFs in fibroblasts co-expressing AcGFP1-actin and zyxin-TagRFP (Fig. 2A). As shown in Fig. 2B,C, zyxin rapidly accumulated at the damaged sites on SFs and then dissociated as repair progressed. These observations were in agreement with the previous observation by Smith et al., suggesting that similar repair mechanisms were at work.

Zyxin is widely recognized as a mechanosensory protein because its intracellular localization changes in response to various mechanical signals. The application of cyclic stretch and sheer stress to cultured cells, which mimic pulse beat and blood flow on vessel walls, respectively, induced zyxin translocation from FAs to SFs (Yoshigi et al., 2005). A stiff substrate for cells enhances zyxin flow from FAs along SFs and causes the thickening of SFs (Guo and Wang, 2007). Changes in intracellular mechanical properties also affect zyxin localization. For example, reduction of the internal tensile force on SFs by myosin II inhibition diminished zyxin accumulation and actin polymerization at FAs (Hirata et al., 2008). Furthermore, local reduction of tension on SFs induced by myosin II or artificial manipulation stimulated the translocation of zyxin to the damaged sites on SFs (Smith et al., 2010; Colombelli et al., 2009). The mechanism by which zyxin translocation is regulated, however, has not been clarified.

### Transient paxillin accumulation at damaged sites on SFs

We searched for other candidate protein(s) involved in the repair of SFs that transiently accumulate at damaged sites because it is highly unlikely that zyxin alone can repair the damaged SFs. It has already been shown that the FA protein, vinculin, is an unlikely candidate (Smith et al., 2010). It is, however, still possible that other candidates may be found among FA proteins.

**Fig. 3.**
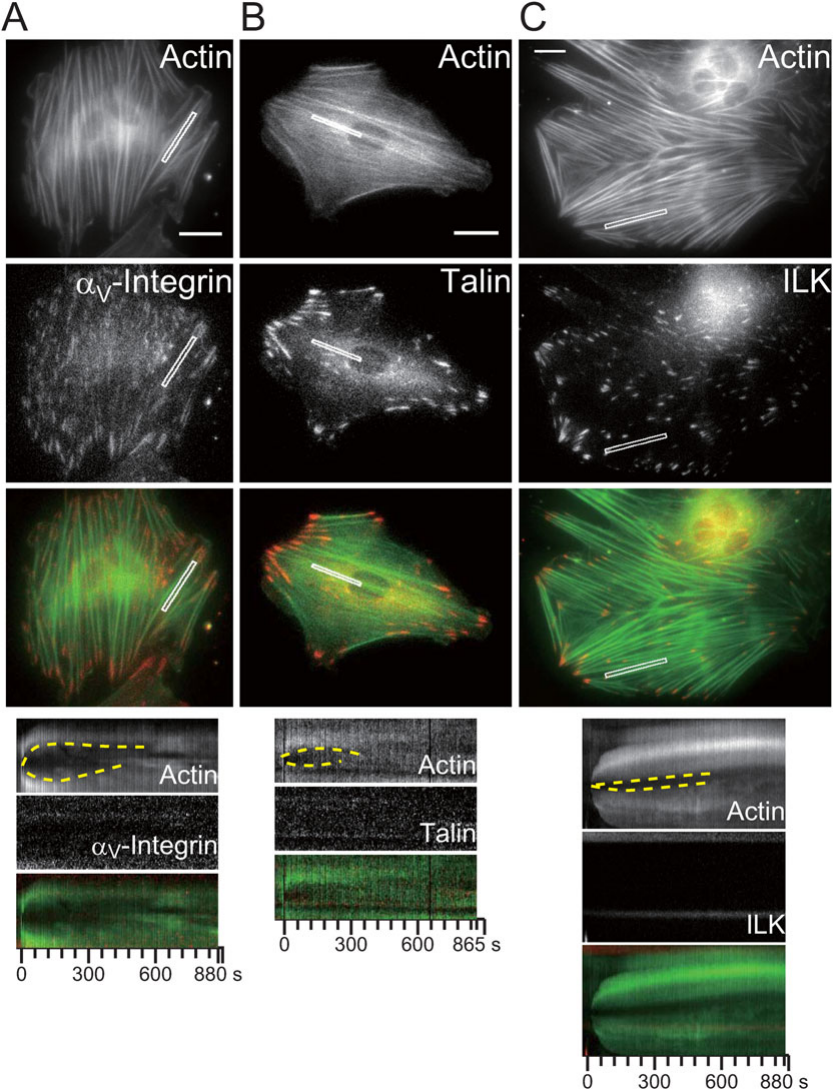
The protein complex which repairs damaged SFs is not a novel focal adhesion. The micrographs and kymographs are of AcGFP1-actin and TagRFP-focal adhesion proteins in rat fibroblasts (**A**: α_V_-integrin, **B**: talin, **C**: ILK). The kymographs (bottom) were obtained from the white boxes in the micrographs. The dashed yellow lines in the kymographs show the strained SFs undergoing thinning, elongation, and then repair. Scale bars: 20 µm.

We prepared fibroblasts that co-expressed AcGFP1-actin and each of the following TagRFP-fused FA proteins, α_V_-integrin, talin, paxillin, or integrin-linked kinase (ILK), to trace whether all, any or none of them accumulated at mechanically-damaged sites on SFs. The results indicated, first, that neither α_V_-integrin nor talin, both of which are structural mediators between ECM and SFs, were recruited to damaged SFs (Fig. 3A,B). Second, we found that paxillin transiently accumulated on damaged SFs, suggesting that paxillin is involved in the SF repair system (Fig. 2D,E,F). Paxillin is a component of FA scaffolds, which provide a platform for several structural and signaling proteins in FAs (Deakin and Turner, 2008). Paxillin accumulated slightly earlier than zyxin, as observed during FA formation (Zaidel-Bar et al., 2003) (Fig. 2G,H). In the case of FA formation, zyxin recruitment was dependent on the presence of IPP complexes composed of ILK, PINCH and α-parvin, whereas paxillin directly interacted with IPP complexes but its localization was not affected by ILK knockout (Stanchi et al., 2009). We examined whether ILK also transiently translocated to damaged SFs and found that ILK was not recruited to the damaged sites during SF repair (Fig. 3C), suggesting that the recruitment of zyxin during SF repair is different to its recruitment during FA formation.

### Transient zyxin accumulation kinetics at damaged SFs was independent of paxillin

**Fig. 4.**
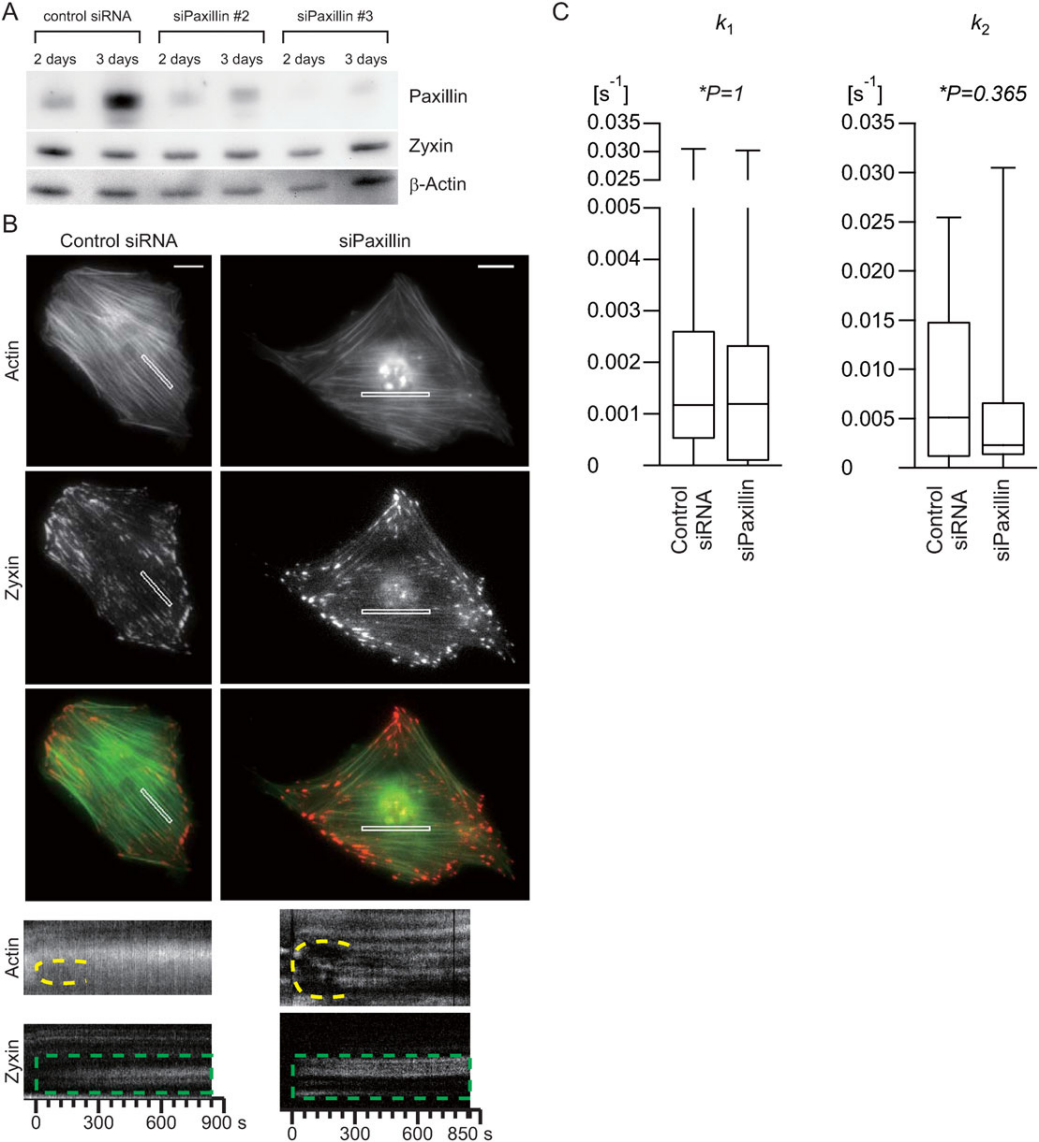
Zyxin accumulation at damaged SFs in paxillin knockdown cells. (**A**) Immunoblot analysis shows paxillin knockdown in the rat fibroblast cell line 2 and 3 days after transfection of rat paxillin siRNAs. (**B**) The micrographs and kymographs are of AcGFP1-actin and zyxin-TagRFP in rat fibroblasts three days after co-transfection of siRNAs for paxillin knockdown or control siRNA. The kymographs (bottom) were obtained from the white boxes in the micrographs and clearly show transient zyxin accumulation after the application of mechanical damage to SFs (dashed green boxes). The dashed yellow lines in the kymographs show the strained SFs undergoing thinning, elongation, and then repair. Scale bars: 20 µm. (**C**) Distributions of the kinetic parameters for zyxin accumulation and dissociation during repair of damaged stress fibres in paxillin knockdown cells as box plots created as described in Fig. 2. All parameters were estimated from the kymographs (control siRNA: 9 cells, 12 SFs; siPaxillin: 13 cells, 21 SFs).

To further clarify the relationship between the transient accumulation of zyxin and paxillin at damaged SFs, we prepared paxillin-knockdown cells co-expressing AcGFP-actin and zyxin-TagRFP and determined the kinetics of zyxin accumulation and dissociation on damaged SFs. Paxillin was knocked down by transforming cells with specific siRNAs, which reduced the paxillin expression level to 11–28% of the control siRNA-transfected cells (Fig. 4A) within three days, while zyxin-TagRFP still localized to FAs in the paxillin knockdown cells (Fig. 4B).

Interestingly, even in paxillin knockdown cells, zyxin accumulated at damaged SFs (Fig. 4B) and partially repaired the damaged fibres. Furthermore, the zyxin accumulation and dissociation rates were not significantly affected by paxillin knockdown (Fig. 4C). Consequently, these results suggested that either zyxin recruitment at damaged SF sites is independent of paxillin or the compensatory pathway may be operating.

### LIM domains in the C-terminus of paxillin are responsible for the transient accumulation of paxillin at damaged SFs

**Fig. 5.**
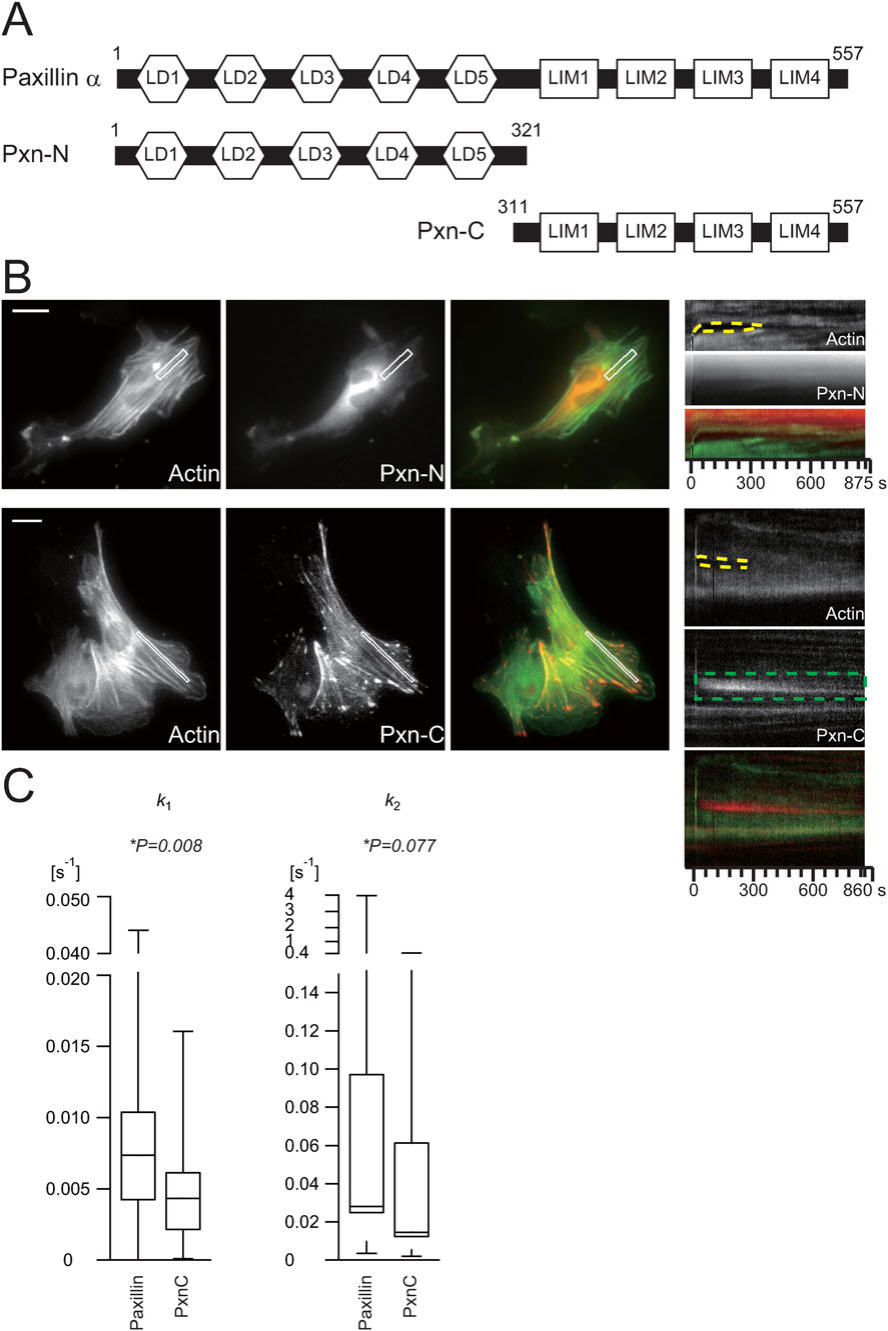
LIM domain requirement for transient paxillin accumulation at damaged SFs. (**A**) Domain structure of paxillin. Paxillin is a 557-amino acid (human), 68-kDa protein comprising multiple structural domains including 5 leucine-rich LD motifs and 4 double zinc finger LIM domains. The deletion mutants lack either the 5 LD motifs (Pxn-C) or the 4 LIM domains (Pxn-N). (**B**) The micrographs and kymographs are of AcGFP1-actin and TagRFP-Pxn-N or TagRFP-Pxn-C in rat fibroblasts. Scale bars: 20 µm. (**C**) Distributions of the kinetic parameters for accumulation and dissociation of full length paxillin and Pxn-C during repair of damaged stress fibres as box plots created as described in Fig. 2. All parameters were estimated from the kymographs (full length paxillin: 16 cells, 33 SFs; Pxn-C: 13 cells, 35 SFs).

To clarify the molecular basis of paxillin localization, we examined the effect of deleting specific regions in paxillin on its ability to transiently translocate to damaged SFs. In Fig. 5A, schematic structures of full length paxillin and the deletion mutants used in this study are shown. The N-terminal half of paxillin includes five leucine- and aspartate-rich LD motifs (LD1–LD5) containing the consensus sequence, LDxLLxxL, which provide protein-binding modules for FAK, vinculin, ILK etc. (Deakin and Turner, 2008; Tumbarello et al., 2002) and are thought to be responsible for most of the signaling functions of paxillin. The C-terminal half of paxillin is composed of four LIM (Lin11, Isl-1, Mec-3) domains (LIM1–LIM4), which form double-zinc-finger motifs that mediate specific protein–protein interactions (Pérez-Alvarado et al., 1994; Schmeichel and Beckerle, 1994). The LIM2 and LIM3 domains in paxillin are essential for targeting paxillin to FAs (Brown et al., 1996), but docking proteins for paxillin remain unidentified.

We observed the behavior of the paxillin deletion mutants after applying damage to SFs in fibroblasts co-expressing AcGFP1-actin and TagRFP-fused paxillin mutants, the latter lacking either the C-terminus (Pxn-N) or the N-terminus (Pxn-C). Consistent with previous work (Brown et al., 1996), Pxn-N failed to localize to FAs. In contrast, Pxn-C did localize to FAs (Fig. 5B) and also transiently accumulated at damaged SFs, but to a lesser extent and at a decreased rate compared with full length paxillin (Fig. 5C). These results suggested that the LIM domains in paxillin play a crucial role in its transient translocation in response to mechanical cues. The N-terminal half of paxillin also contributes to the transient accumulation kinetics of paxillin. This finding raises interest in the universal roles of LIM domains in cellular mechanosensory systems. Zyxin contains three C-terminal LIM domains that are crucial for its force-induced accumulation at FAs (Uemura et al., 2011; Steele et al., 2012). Hic-5, an FA protein of the paxillin family, contains four LIM domains at its C-terminus (Brown and Turner, 2004) and shows restricted expression in mononuclear smooth muscle cells in adult humans, whereas paxillin is widespread (Yuminamochi et al., 2003). Interestingly, while zyxin and Hic-5 translocate to SFs in response to cyclic stretch (Yoshigi et al., 2005; Kim-Kaneyama et al., 2005; Shikata et al., 2005), paxillin remains localized at FAs without translocating to SFs, suggesting that the response mechanism to cyclic stretch may be different from the response to local SF damage. Indeed, zyxin translocation to SFs from FAs in response to cyclic stretch depends on integrin function, whereas the response to SF damaged sites is independent of FA formation via integrins (Smith et al., 2010; Yoshigi et al., 2005) (Fig. 3). The docking sites in SFs and the regulation of the transient translocation of these LIM proteins during SF repair are key issues to be resolved in future studies.

Our data highlight the role of paxillin as a major mechanosensor for the maintenance of locally damaged SFs. Paxillin rapidly translocated to SFs slightly earlier than zyxin, which is quite different to the mechanism of FA formation. The C-terminal LIM domains in paxillin are sufficient to mobilize paxillin to damaged SFs. Identification of mediators between SFs and LIM domains in paxillin and zyxin will contribute to our understanding of the role of the mechanotransduction system in maintaining intracellular mechanical homeostasis.

## Materials and Methods

### DNA constructs

The pAcGFP1-MCLinker-actin plasmid was prepared by replacing the humanized monomeric Kusabira Orange-1 (hmKO1)-coding region in phmKO1-MCLinker-actin (Watanabe-Nakayama et al., 2010) with the AcGFP1-coding region from pAcGFP1-actin (Clontech) using the *Mro* I (Toyobo) and *Bsh* TI (Fermentas) restriction enzymes. The mammalian expression vectors, pTagRFP-MNLinker and pTagRFP-MCLinker, were prepared by replacing the hmKO1-coding regions in phmKO1-MNLinker and phmKO1-MCLinker (MBL) with TagRFP-coding fragments generated by PCR and excised from pTagRFP-actin (Evrogen) with *Bsh* TI and *Xba* I (Fermentas). The cDNA fragments, including full-length human α_V_-integrin and human zyxin, were inserted into the pTagRFP-MNLinker vector, while full-length rat talin, human paxillin and rat ILK were inserted into the pTagRFP-MCLinker vector.

### Cell culture and transfection of plasmid DNA

A fibroblastic cell line cloned from the rat vomeronasal organ (VNOf) was used throughout this study mainly because these cells are large enough for AFM manipulation of their SFs under the optical microscope. The cells were maintained as described in the previous work (Watanabe-Nakayama et al., 2011). Transfection was performed by electroporation using a Neon transfection system (Invitrogen). After electroporation, the cells were spread onto 22 mm diameter glass slips (Matsunami Glass Ind.) coated with fibronectin (CalBioChem) and maintained in Leibovitz's L-15 medium supplemented with 10% fetal bovine serum. For AFM experiments, the glass slips were inserted into the BioCell module of the Nanowizard II AFM (JPK Instruments) and maintained at 37°C.

### Application of external force

The NanoWizard II AFM (JPK Instruments) mounted on an Axio-Observer D1 inverted microscope (Carl Zeiss) was used for all manipulations and optical observations (Hakari et al., 2011). ATEC-CONT arrow-head cantilevers (Nanosensors) were used for manipulation because their protruded tip is easily visualized when mounted onto the AFM head. The sensitivity of the optical lever system was calibrated and the cantilever spring constant was determined in situ before each experiment by the thermal noise method (Hutter and Bechhoefer, 1993; Butt and Jaschke, 1995). SF manipulation experiments were performed at 37°C using a temperature-controlled BioCell (JPK Instruments). SF manipulation was performed by moving the AFM cantilever horizontally at a speed of 1 µm/s to push the cell surface. The cantilever position and its vertical and lateral deflection were recorded during the manipulation.

### Image acquisition and analysis

Imaging was performed on the inverted microscope described above, using a back-illuminated electron-multiplying charge-coupled device camera (model iXon X3 DU897E-CS0-#BV; Andor Technology) and a ×40 objective lens (Carl Zeiss). Illumination was obtained from 470 nm and 565 nm light emitting diode array modules installed in a CoolLED pE-2 excitation system (CoolLED). The emission light path was equipped with a dual bandpass filter (GFP/DsRed-A; Semrock Inc) and single band path filters (GFP-3035B and TRITC-B; Semrock Inc) for live cell imaging. All time-lapse image sequences were captured at 2.5 s or 5 s intervals. Image acquisition was performed using Andor IQ2 imaging software (Andor Technologies).

For preparation of kymographs and time analysis of the evolution of fluorescence intensity, image sequences were processed using Image J software. The background fluorescence level was subtracted from the time-lapse images using a rolling ball background subtraction algorithm in Image J, and then corrected for photobleaching using a single exponential and a double exponential function described in the literature (Vicente et al., 2007). Kymographs were generated from the processed images using a general command (Reslice) in Image J as follows: an *x-t* line scan image volume (kymograph volume) was reconstructed along the longitudinal axis of regions of interest (ROI) (along a target SF). The image volume slices were stacked to create a cumulative image in which the fluorescence intensity of the ROI in each time period (frame) was represented by a 1 pixel width for all experiments.

Time evolution of zyxin and paxillin accumulation was fitted with a kinetic equation for a successive reaction as follows:
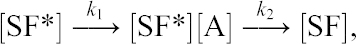
where SF*, SF and A correspond to a damaged SF, a repaired SF, and a repair-promoting protein such as zyxin or paxillin, respectively. *k*_1_ and *k*_2_ are the association and dissociation rate constants, respectively. Evolution and decay of the intermediate, [SF*][A], was quantified as the change in the fluorescence level of zyxin or paxillin. Therefore, the time evolution of fluorescence intensity, F.I., for zyxin or paxillin on damaged SFs can be fitted with the following equation:
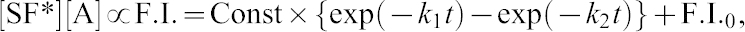
where F.I._0_ corresponds to the offset level. The values of *k*_1_ and *k*_2_ that gave the best fits to the experimental results are shown in box plots in the figures.

### RNAi

VNOf cells were transiently transfected with Stealth RNAi siRNA oligonucleotide duplexes (Invitrogen) for RNAi experiments. The target sequences were selected from two different regions within rat paxillin mRNA: 5′-CCCACAUCUCCAAACGGCCAGUGUU-3′ and 5′-CAGGACAGUGUCGGCUCCCUUUGUU-3′. As a negative control, a high GC duplex from the Stealth RNAi negative control duplexes (Invitrogen) was used three days after transfection.

### Antibodies

Rabbit polyclonal antibodies to paxillin (H-114) and zyxin (ab71842), which were used for immunoblotting, were obtained from Santa Cruz Biotechnology and Abcam, respectively. Mouse monoclonal antibody to β-actin (clone 6D1) purchased from MBL.

### Immunoblot analysis

Cell lysates were prepared in SDS-PAGE sample buffer (62.5 mM Tris-HCl, pH 6.8, 100 mM dithiothreitol (DTT), 2% SDS, 8.7% glycerol and bromophenol blue) and subjected to SDS-PAGE followed by immunoblot analysis (Phast system; GE Healthcare). For immunoblot analysis, proteins were transferred electrophoretically from the gels to polyvinylidene difluoride membranes (Hybond LFP; GE Healthcare), which were then incubated for 1 h at room temperature with ECL prime blocking reagent (GE Healthcare) to block nonspecific sites. The membranes were probed with primary antibodies, washed three times, and incubated with horseradish peroxidase-conjugated secondary antibodies. Immune complexes were visualized using an enhanced chemiluminescence kit (ECL Prime; GE Healthcare).
